# In silico drug repurposing for filarial infection predicts nilotinib and paritaprevir as potential inhibitors of the *Wolbachia* 5′-aminolevulinic acid synthase

**DOI:** 10.1038/s41598-021-87976-4

**Published:** 2021-04-19

**Authors:** Alexander Kwarteng, Ebenezer Asiedu, Augustina Sylverken, Amma Larbi, Yusif Mubarik, Charles Apprey

**Affiliations:** 1 Department of Biochemistry and Biotechnology, Kwame Nkrumah University of Science and Technology, KNUST, Kumasi, Ghana; 2 Kumasi Centre for Collaborative Research in Tropical Medicine, Kwame Nkrumah University of Science and Technology, KNUST, Kumasi, Ghana; 3 Department of Theoretical and Applied Biology, Kwame Nkrumah University of Science and Technology, KNUST, Kumasi, Ghana

**Keywords:** Computational biology and bioinformatics, Drug discovery

## Abstract

Filarial infections affect millions of individuals and are responsible for some notorious disabilities. Current treatment options involve repeated mass drug administrations, which have been met with several challenges despite some successes. Administration of doxycycline, an anti-Wolbachia agent, has shown clinical effectiveness but has several limitations, including long treatment durations and contraindications. We describe the use of an in silico drug repurposing approach to screening a library of over 3200 FDA-approved medications against the filarial endosymbiont, *Wolbachia*. We target the enzyme which catalyzes the first step of heme biosynthesis in the *Wolbachia*. This presents an opportunity to inhibit heme synthesis, which leads to depriving the filarial worm of heme, resulting in a subsequent macrofilaricidal effect. High throughput virtual screening, molecular docking and molecular simulations with binding energy calculations led to the identification of paritaprevir and nilotinib as potential anti-Wolbachia agents. Having higher binding affinities to the catalytic pocket than the natural substrate, these drugs have the structural potential to bind and engage active site residues of the *wolbachia* 5′-Aminolevulinic Acid Synthase. We hereby propose paritaprevir and nilotinib for experimental validations as anti-Wolbachia agents.

## Introduction

Filarial infections are classified as neglected tropical diseases (NTDs), and they present severe health threats to affected populations. Filarial infections are endemic in some African countries and parts of Latin America, with over several billions of individuals at risk of these infections^1,2^. Several filarial nematodes cause the disease; *Wuchereria bancrofti*, *Brugia malayi and Brugia timori* (lymphatic filariasis)*, Onchocerca volvulus* (river blindness)*, Mansonella sp.* (Mansonellosis) and *Loa loa* (loaisis).

Currently, filarial infections are managed with mass drug administration (MDA) programs and vector control practices. The MDA programs involve periodic administration of ivermectin, diethylcarbamazine (DEC), and albendazole, which aim to interfere with the developmental cycle of the nematodes by killing the microfilariae. However, the treatment by these MDA agents is known to partially affect the adult worms and thus, may continue to reproduce microfilariae^2–4^. Moreover, there are serious complications after the administration of these drugs in filarial co-endemic areas. For example, individuals co-infected with loiasis develop encephalopathy and may even die when treated with ivermectin or diethylcarbamzine^3,5^.

Most causative agents of filarial infections harbor intracellular bacteria known as *Wolbachia*^6–8^. The *Wolbachia* has a symbiotic relationship with the filarial worms that involves several benefits to the nematode^6,7^. The bacteria endosymbionts also play roles in the development of filarial pathologies such as lymphoedema and hydrocele^6,8^. The *Wolbachia* trigger host’s innate immunity via macrophage activating neutrophils and toll-like receptor 2 (TLR-2) pathway, resulting in the release of pro-inflammatory cytokines such as interleukin-6 (IL-6) and tumor necrosis factor-alpha (TNF-α)^9–11^. High levels of these cytokines activate vascular endothelial growth factor pathways, which is a key player in the development of filarial pathologies^9–11^.

Lately, the *Wolbachia* endosymbiont of filarial worms has been an attractive therapeutic target for managing filarial infections. Anti-*Wolbachia* drugs such as doxycycline and rifampicin have been demonstrated to have suicidal effects on adult worms in both lymphatic filariasis and onchocerciasis^12–14^. Furthermore, since *Loa loa* lacks the *Wolbachia* endosymbiont^15^, using anti-Wolbachia drugs prevents the complications (encephalopathy or even death) associated with the treatment of *Loa loa* co-infected individuals. These anti-Wolbachia drugs, although effective, have several limitations that justify the need for novel drugs to be used together with the MDA regimens or replace current treatment options. For instance, doxycycline, requires long treatment regimens of 4–6 weeks, which presents certain logistical constraints to its use on larger populations^12,13^. There is an urgent need for improved treatment options against filaria infections, considering the many challenges associated with current therapeutics. Modification of the treatment durations for shorter time intervals is being studied by the Anti-Wolbachia (A-WOL) consortium (https://awol.lstmed.ac.uk/).

The heme biosynthesis pathway of the *Wolbachia* endosymbiont is one of the most important biochemical pathways in the symbiotic relationship between the worm and the *Wolbachia*^16,17^. Heme is a co-factor for several proteins, including hemoglobin, catalase, and peroxidase, required for many vital biological processes. Comparative genomics studies have revealed that most nematodes are incapable of the *de-novo* synthesis of heme, as they lack vital genes involved in the heme biosynthesis pathway^16^. These observations imply that filarial worms acquire heme biosynthesis products or its intermediates from the intracellular *Wolbachia* for consumption or salvage synthesis. Recent experimental data demonstrate that the genes responsible for heme biosynthesis in *Wolbachia* are crucial to the survival of the filarial host^16^. The heme biosynthesis genes of the *Wolbachia* deviate largely from their counterparts in humans in terms of phylogeny and exhibit significant differences in sensitivity to the heme pathway inhibitors, making the heme pathway an ideal therapeutic target for filaria-borne diseases. 5′-Aminolevulinic Acid Synthase (ALAS) is the first enzyme in the heme biosynthesis pathway, which catalyzes the production of 5′-aminolevulinate from glycine and succinyl-coenzyme A (S-CoA) using pyridoxal 5′-phosphate (PLP) as a co-factor^18–20^.

Repurposing of drugs refers to the re-orientation of approved or investigational drugs for new therapeutic roles. This strategy has several advantages over the traditional drug discovery routine, given that the drug candidates have clinical profiles and pharmacological profiles documented and validated^21^. Drug repurposing is less expensive, has a shorter development duration and a lower risk of failure. The in silico-based drug repurposing approach has been an important aspect of drug discovery programs adopted by many research groups and pharmaceutical institutions^21^. Compared to the activity-based approach of drug repurposing, the in silico-based approach is time and labor efficient^22^.

**Figure 1:**
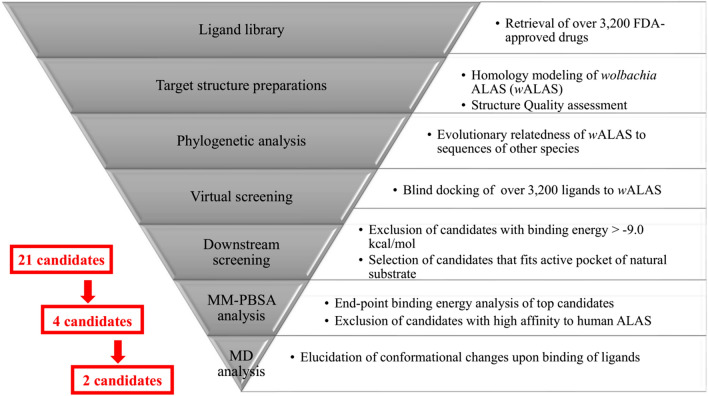
Flowchart depicting the in silico drug repurposing approach for the study.

In this study, we used in silico drug repurposing approach to discover potential anti-Wolbachia drug candidates as therapeutic options for filarial infections (Fig. 1). We have identified paritaprevir and nilotinib, both FDA-approved medications for managing chronic Hepatitis C and leukemia respectively, as potential anti-Wolbachia agents. These drugs have the structural potential to bind and engage active site residues of *wolbachia* 5′-Aminolevulinic Acid Synthase (*w*ALAS), the first enzyme of heme biosynthesis in *Wolbachia*.

## Methods

### Preparation of ligand library

The ligand library comprised of FDA-approved drugs was obtained from the ZINC database^23^ (http://zinc15.docking.org/). A total of 3210 compounds classified as FDA-approved were retrieved from the database as structure data files (*sdf* formats). Refinements of the ligand library were performed by PyRx *v*0.8^24^. The refinement process involved ligand inter-conversion and ligand minimization. The sdf files were energy minimized with *Amber force field* and converted to autodock compatible file formats (*pdbqt*) using Open Babel^25^. The conversion involved the addition of Gasteiger partial charges and polar hydrogen atoms to the ligands.

### Target structure preparations

There is currently no reported crystallographic structure for the *Wolbachia* ALAS (*w*ALAS), although the protein sequence data is available at the UniProt database (https://www.uniprot.org/uniprot/A0A225X627). The protein sequence of the *Wuchereria bancrofti* endosymbiont ALAS was obtained from UniProt. The structure of *w*ALAS was accordingly generated computationally using its protein sequence and the crystallographic co-ordinates of *Rhodobacter capsulatus*^18^ (PDB ID: 2BWP), courtesy of the SWISS-MODEL tool^26^. Structure quality assessment of the protein models was also performed by the SWISS-MODEL quality assessment tool (https://swissmodel.expasy.org/assess). All visualization and interaction analyses were performed with Pymol Molecular Visualization Software *v*2.4^27^.

### Phylogenetic analysis

The Clustal omega tool was used to determine the evolutionary relatedness of *w*ALAS to other filarial nematodes and other species. The server (http://consurf.tau.ac.il) provides an evolutionary profile of the amino acids, which define their level of importance to the protein’s biological activity and structure. The following parameters were selected for phylogenetic analysis: homologous search algorithm: CSI-BLAST; number of iterations: 3; E-value cut-off: 0.0001; protein database: UNIREF-90; number of reference sequences selected: 150; maximum sequence identity: 95%; minimum identity for counterparts: 35%; alignment method: Bayesian; calculation method: MAFFT-L-INS-i; and evolutionary substitution model: best model.

### Virtual screening

The virtual screening was performed with PyRx *v*0.8^24^. We employed the AutoDock Vina^28^ Lamarckian Genetic algorithm and Empirical Free Energy Scoring function within the PyRx v0.8 interface. All 3210 prepared ligands were targeted against the *w*ALAS protein in a blind docking manner. The grid for the target protein was set to 126 Å by 126 Å by 126 Å with a spacing of 1.000 Å. A total of 8 different poses were generated for each ligand and the lowest energy poses were considered. All graphs of docking results were plotted with GraphPad *v*9.0.

### MM-PBSA analysis

The binding energy of the ligands was calculated using the Molecular Mechanics with Poisson–Boltzmann Surface Area (MM-PBSA) analysis as described previously^29^. A short molecular simulation of the complexes (10 ns) was performed with GROMACS *v*5.5.2^30^ following the protocol described in the section below, and the trajectories were used for the calculations. The binding energy (E_binding_) of the system is estimated as;

$${\text{E}}_{{{\text{binding}}}} \, = \,{\text{E}}_{{{\text{complex}}}} \, - \,\left( {{\text{E}}_{{{\text{target}}}} \, + \,{\text{E}}_{{{\text{ligand}}}} } \right)$$

where E_complex_ is the total free energy of the target-ligand complex, E_target_ and E_ligand_ are total free energies of the individual target (receptor) and ligand in a solvent, respectively. The individual binding free energy of each component is expressed as;

$${\text{E}}_{{{\text{binding}}}} \, = \,{\text{E}}_{{{\text{MM}}}} \, + \,{\text{G}}_{{{\text{solv}}}}$$

where E_mm_ represents the molecular mechanics energy terms, G_solv_ represents the solvation energy terms. It is worth noting that the entropic term (ΔTS) is exempted from the calculation, particularly due to the high computational demand and there are reports demonstrating that the net contribution of the entropic term is often minimal^29^. This is why the binding energy is designated as E_binding_ instead of ΔG. The E_mm_ is made up of all bonded and non-bonded energies in the system, thus, can be expressed as;

$${\text{E}}_{{{\text{MM}}}} \, = \,{\text{E}}_{{{\text{bonded}}}} \, + \,{\text{E}}_{{{\text{nonbonded}}}} \, = \,{\text{E}}_{{{\text{bonded}}}} \, + \,{\text{E}}_{{{\text{vdW}}}} \, + \,{\text{E}}_{{{\text{elec}}}}$$

where E_bonded_ is the bonded interactions consisting of bond, angle, dihedral and improper interactions. E_nonbonded_ represents the non-bonded interactions that include both electrostatic (E_elec_) and van der Waals (E_vdW_) interactions, which are calculated using Coulomb and Lennard–Jones potential functions, respectively. The solvation energy term (G_solv_) is expressed as:

$${\text{G}}_{{{\text{solv}}}} \, = \,{\text{G}}_{{{\text{polar}}}} \, + \,{\text{G}}_{{{\text{nonpolar}}}}$$

where G_polar_ represents polar solvation energies and G_nonpolar_ is the non-polar solvation energies. G_polar_, which is the electrostatic contribution, is calculated from solving the Poisson-Boltzmann equation. The non-electrostatic term of solvation energy, G_nonpolar_, includes repulsive and attractive forces between solute and solvent generated by cavity formation and van der Waals interactions, respectively^29^.

### Molecular dynamics simulation

Molecular dynamics simulation of the complexes was performed using GROMACS 2020.3^30^ and CHARMM36 force field^31^. The molecular systems were centred in a cubic box and solvated with three-point (tip3p) water model. The systems were neutralized with NaCl before 10,000 steps energy minimization using the steepest descent algorithm and maximum force threshold of 100 kJ/mol/nm. Van der-Waals interactions were treated with a single cut-off of 1.4 nm. Long-range electrostatics were treated with the Particle-Mesh Ewald (PME) method with a 0.168 fast Fourier transform (FFT) grid spacing and 4th order B-spline interpolation and a cut-off of 1.4 nm. Neighbor search was performed every 20 steps using the grid method with Verlet cut-off scheme. Protein and non-protein components of the system were independently coupled to v-rescale thermostat and an isotropic Berendsen algorithm for pressure coupling. All bonds within the protein were constrained using LINCS algorithm. The system was equilibrated by maintaining a constant temperature and pressure for 400 ps. The *w*ALAS protein system was simulated for 500 ns and the *w*ALAS-ligand complexes were simulated for 200 ns. All graphic representations of simulation trajectories were generated with the GRACE plotting tool (http://plasma-gate.weizmann.ac.il/Grace). In-built GROMACS tools were used for all trajectory processing, including re-centering, fitting, and periodicity treatments before subjected to analysis. The dynamics of the molecular systems were evaluated based on properties such as root-mean-square deviation (RMSD), root-mean-square fluctuation (RMSF), gyration radius (Rg), and hydrogen bonds. RMSD was calculated overall backbone atoms after least-square fitting to the reference backbone, while the RMSF was calculated *per* residue after least-square fitting to backbone atoms.

### Clustering of structures

The simulation produces several structural frames that can be clustered based on the structural deviations from the reference structure. The backbone atoms of the protein structures were used for the structure superposition and clustering. We used the *gromos* method^32^ and a clustering RMSD cut-off of 0.10 nm. The algorithm counts the number of neighbours using the RMSD cut-off, takes the structure with the largest number of neighbours with all its neighbours as cluster and eliminate it from the pool of clusters. This is repeated for the remaining structures in the pool. For each trajectory, the middle structure of the top ranked cluster group was selected as the cluster representative and used for further analyses.

## Results

### Structure modeling of *Wolbachia *ALAS (*w*ALAS)

**Figure 2:**
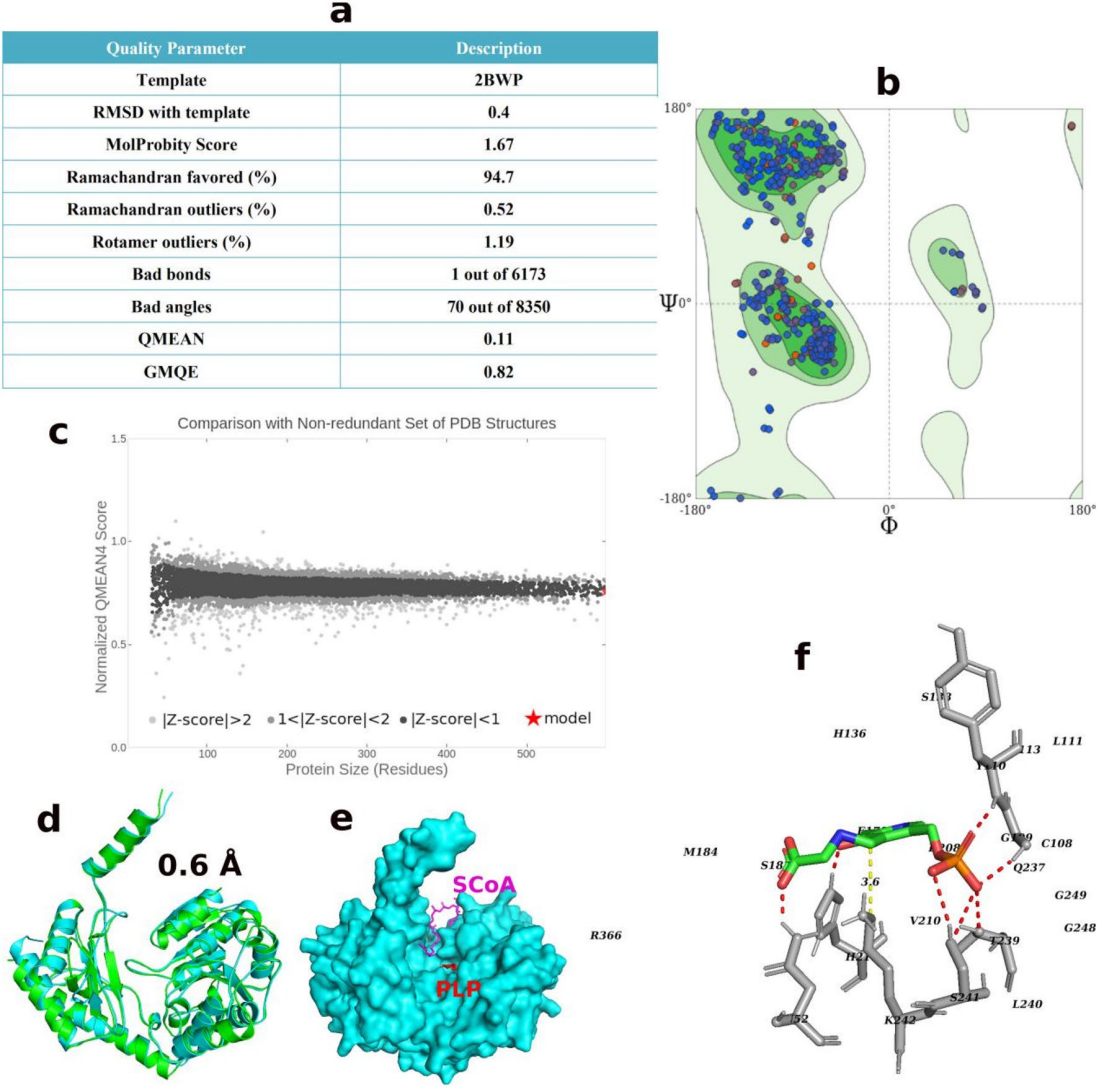
Modeling of *w*ALAS structure. (**a**) Assessment of structure quality for the *w*ALAS model. (**b**) Ramachandran plot of the *w*ALAS homodimer. (**c**) Comparison of model structure to set of non-redundant crystallographic proteins in the protein data bank. (**d**) Structural superposition of the template structure and the model (**e**) Binding pockets of co-factor and ScoA in ALAS (**f**) Active site characterization of PLP-Gly in *w*ALAS. Polar interactions between the molecules are represented as red dashed lines. The distance between the Lys-242 and PLP-Gly is represented as yellow dashed line.

The structure of the *Wolbachia* ALAS (*w*ALAS) was computationally modeled based on the crystallographic coordinates of the *Rhodobacter capsulatus* ALAS (PDB ID: 2BWP)^18^. The quality evaluation of the model was informed from the molprobity score, Ramachandran plot, global model quality estimation (GMQE) and qualitative model energy analysis (QMEAN). The evaluation scores, shown in Fig. 2a, collectively suggest that the protein model has good quality and suitability for downstream analysis. A plot of the *psi* and *phi* angles in the generated *w*ALAS model has been provided in Fig. 2b. The distribution of the residual angles depicted a conformation with less clashes and dominated by β-helices. Moreover, a comparison of the built model with a set of non-redundant protein structures in the PDB also confirmed the *w*ALAS quality (Fig. 2c). The overall structural configuration was highly similar to the template with a root-mean-square deviation (RMSD) of 0.6 Å based on structural superposition (Fig. 2d). *w*ALAS is a homo-dimer with 400 amino acid residues per monomer unit. The catalytic domain lies between the N-terminal domain (NTD) and the C-terminal domain (CTD), with distinct binding pockets for PLP and S-CoA (Fig. 2e). Prior to catalysis, in the absence of substrates, PLP forms a covalent bond with a lysine residue that is believed to be conserved in the active site of all ALAS^18,19^. In the *w*ALAS model, Lys-242 would be the residue to be covalently bound to PLP (Fig. 2f). Lys-242 is appropriately positioned to the PLP with an interval of 3.6 Å (Fig. 2f). During catalysis, PLP loses the covalent bond to Lys-242 to allow the binding of the substrate glycine to the co-factor through a Schiff base linkage, herein referred to as PLP-Gly^18,19^.

We superimposed the template structure (in complex to PLP-Gly) to the built model to characterize the active pocket of *w*ALAS. The active site residues surrounding the PLP-Gly, as present in *w*ALAS, are shown in Fig. 2f. We considered the active site residues to be all amino acids within 5 Å of the ligand, assumption that all relevant interaction forces between the ligand and the protein are captured within 5 Å distance. The activity resulted in 26 amino acid residues in the specified binding pocket; Asn-52, Cys-108, Gly-109, Tyr-110, Leu-111, Asn-113, His-136, Ser-138, Met-139, Glu-179, Ile-181, Tyr-182, Ser-183, Met-184, Asp-208, Val-210, His-211, Gln-237, Thr-239, Leu-240, Ser-241, Lys-242, Gly-248, Gly-249, Thr-357, and Arg-366. Residues involved in polar interaction with PLP-Gly at the active site include Asn-52, Glu-109, Tyr-110, His-211, Thr-239, and Ser-241 (Fig. 2f).

**Figure 3:**
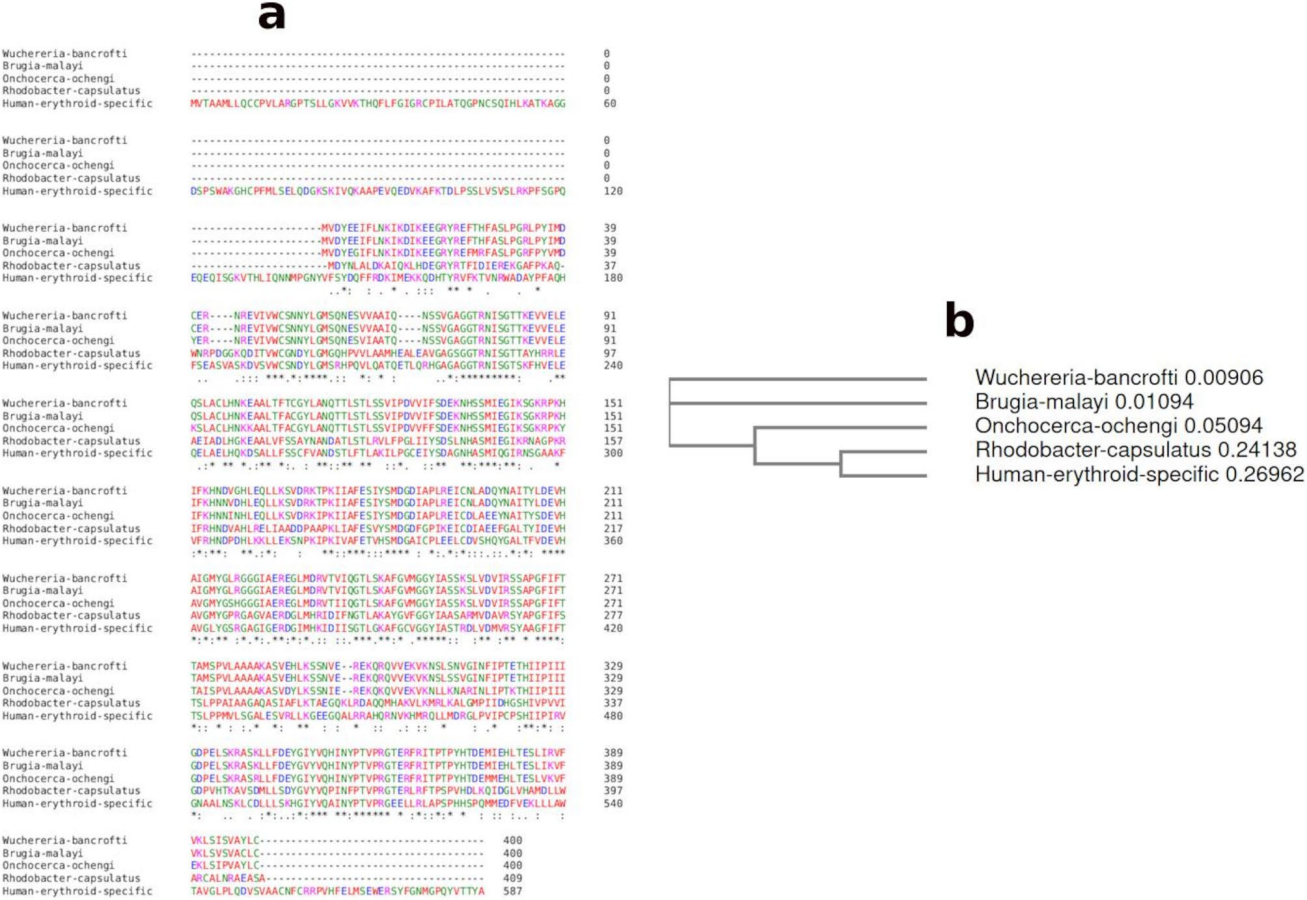
Evolutionary relatedness of *w*ALAS in nematodes and other species. (**a**) Multiple sequence alignment of *w*ALAS of *W. bancrofti* endosymbiont, *B. malayi* endosymbiont, *R. capsulatus* and human. (**b**) Simple phylogram depicting the evolutionary relatedness of the *w*ALAS sequences.

In the present study, we used the protein sequence belonging to the *W. bancrofti*’s endosymbiont, considering that *W. bancrofti* is responsible for 90% of filarial infections^4^. We compared the protein sequence of ALAS in *B. malayi*, *R. capsulatus* and humans to check for evolutionary relatedness (Fig. 3a). The residues of *w*ALAS are highly conserved in *W. bancrofti* and *B. malayi* when compared to corresponding sequences of *R. capsulatus* and humans. The simple phylogram further confirms the evolutionary relatedness of ALAS in the filarial nematodes and the divergence across the four species studied (Fig. 3b). Despite the evolutionary divergence, several protein residues are conserved across the four species examined.

### Simulation of wALAS monomer

**Figure 4:**
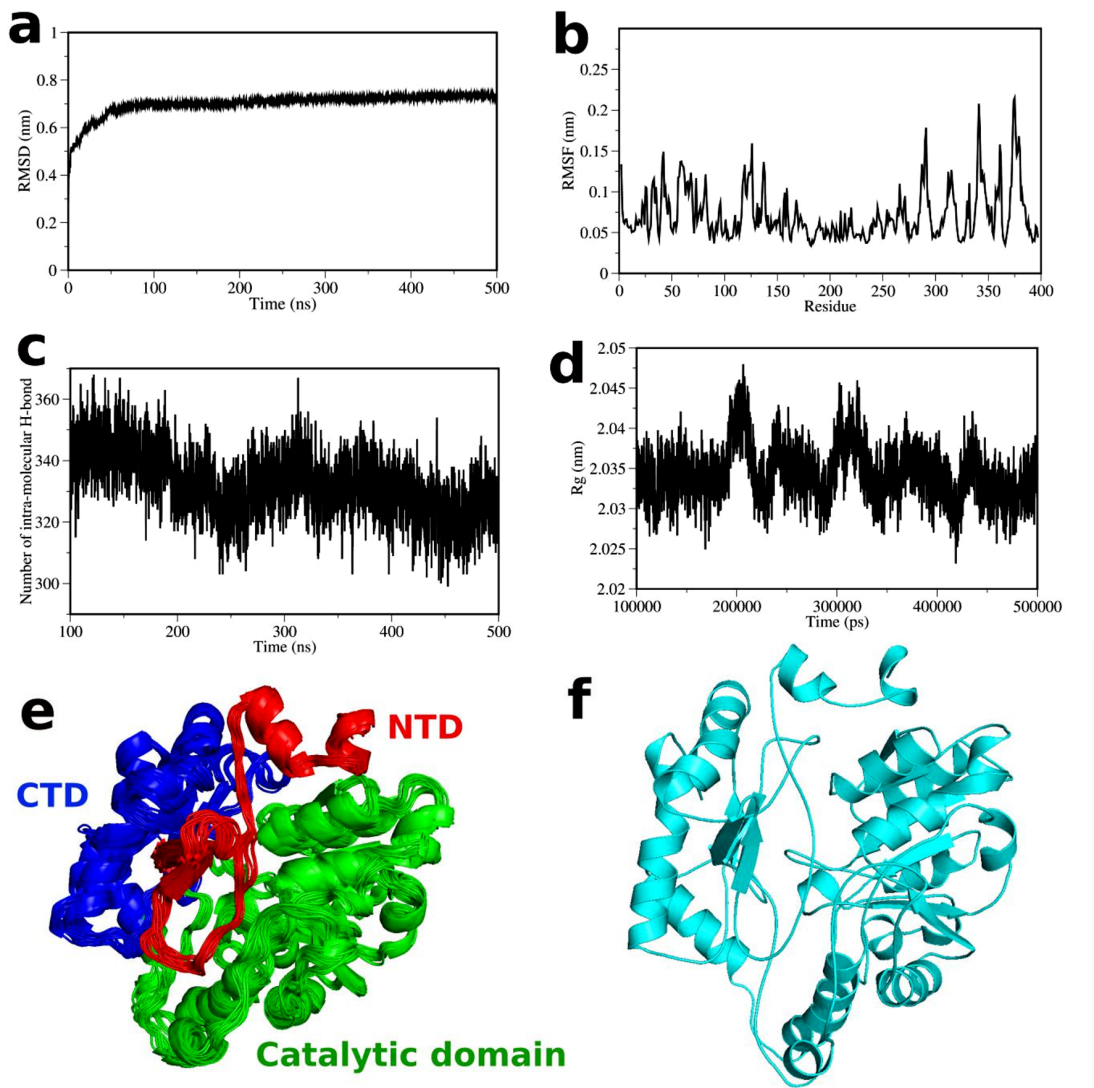
Simulation studies of *w*ALAS monomer. (**a**) Root-mean-square-deviation (RMSD) profile of the *w*ALAS protein over 500 ns. (**b**) Root-mean-square-fluctuation (RMSF) profile of the *w*ALAS protein after least square fit to reference Ca atoms. (**c**) The intramolecular hydrogen bond in the *w*ALAS. (**e**) The average structures of the separate cluster groups showing the catalytic domain and the termini domains. (**f**) The middle structure of the most populated cluster group.

To investigate the structural dynamics of the *w*ALAS protein, we performed a 0.5 μs simulation of the *w*ALAS chain A using GROMACS 2020.3 and CHARMM36 forcefield. The 0.5 μs simulation of the monomeric *w*ALAS seems to be stable with an average RMSD of 0.7 ± 0.04 nm ranging from 0.5 to 0.8 nm (Fig. 4a). Fluctuation of the *w*ALAS residues based on analysis of the last 0.4 μs of the simulation also revealed high residue fluctuation in the NTD and CTD, but relatively lower residue fluctuation in the core of the catalytic domain (Fig. 4b). The compactness of the *w*ALAS monomer as determined by the radius of gyration (Rg) was 2.0 ± 0.003 nm (Fig. 4c). The intra-molecular hydrogen bonds of the *w*ALAS protein was also computed from the last 0.4 μs of the simulation. The number of intra-molecular hydrogen bond in the *w*ALAS averaged 332 ± 11 hydrogen bonds (Fig. 4d). Using the *gromos* method^32^, we clustered the molecular ensembles from the last 0.4 μs of the simulation with 0.1 nm RMSD cut-off. A total of 21 clusters were obtained and the middle structure of each cluster has been shown in Fig. 4e. The most populated cluster among the 21 clusters (Fig. 4f) was used as the representative *w*ALAS structure for the molecular docking studies.

### Screening of FDA-approved drugs: Molecular docking to wALAS

**Figure 5:**
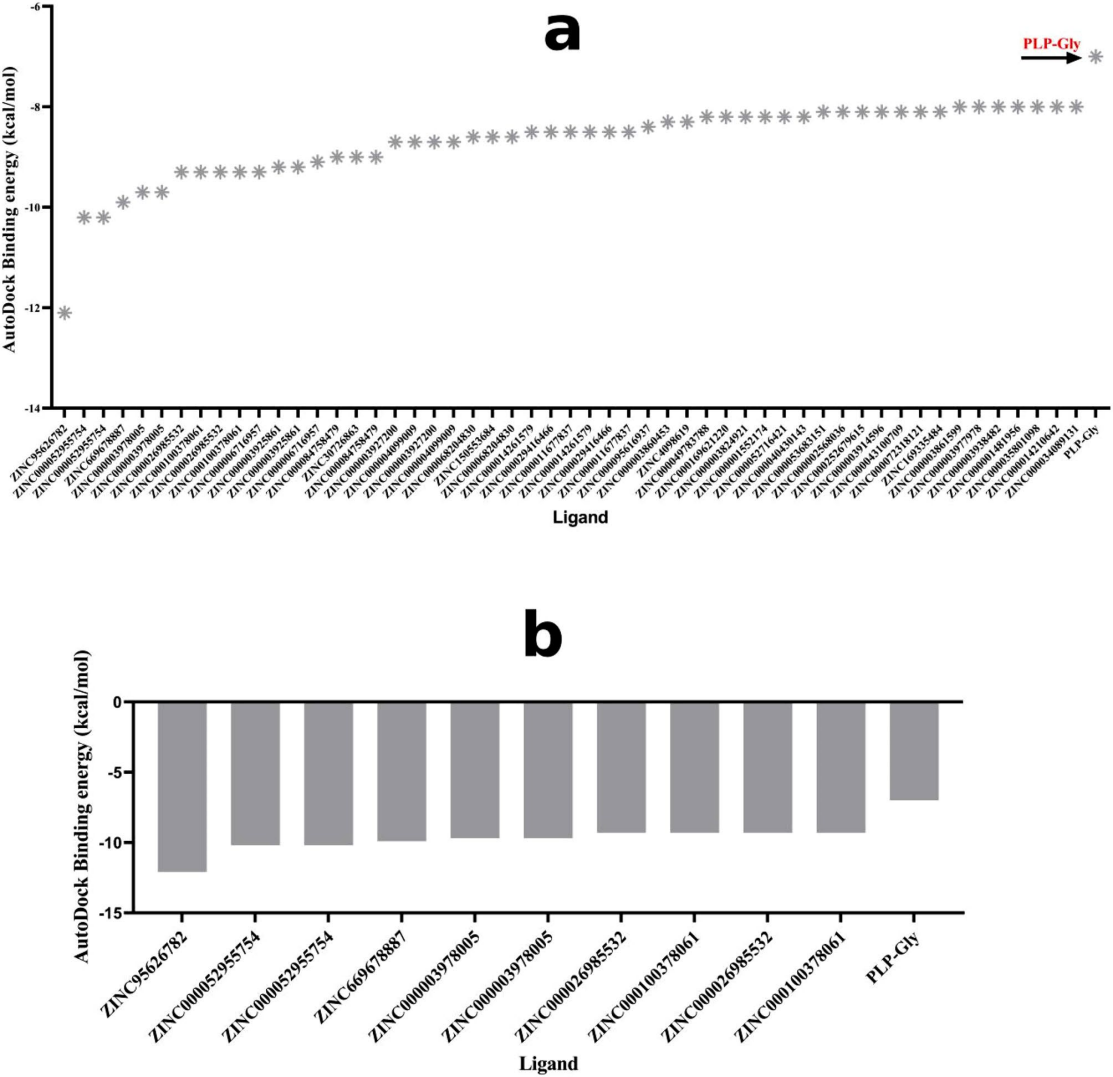
Screening of ligand library against the *w*ALAS. (**a**) Binding energies of the top 50 candidates, compared to PLP-Gly (**b**) The top performing candidates with < − 9 kcal/mol binding energy.

We screened the 3210 FDA-approved drugs from the ZINC15 database^23^ against the *w*ALAS using AutoDock Vina^28^ on PyRx 0.8^24^. The high-throughput virtual screening of the ligand library resulted in a broad range of binding affinity towards the active pocket of *w*ALAS. The natural ligand of *w*ALAS, PLP-Gly, was included in the ligand library as a reference. The AutoDock binding energies of the ligands ranged from − 1.2 to − 12 kcal/mol. Binding energies of the top 50 ligands and PLP-Gly, are shown in Fig. 5a. The autodock binding energy for PLP-Gly was − 7 kcal/mol. From the top 50 performing ligands, we selected candidates scoring − 9 kcal/mol or less, considering the error estimate associated with autodock vina binding energy calculations (± 2 kcal/mol)^28^. The scores of the resulting candidates are shown in Fig. 5b.

The binding site of *w*ALAS has distinct binding pockets to accommodate PLP, glycine and S-CoA. The co-factor of *w*ALAS (PLP) has very crucial roles before and during catalysis. The actual substrate of *w*ALAS, glycine, is properly positioned in the active pocket by PLP through a Schiff base interaction to form an aldimine (PLP-Gly)^18,19^. Similar observations have been made in the ALAS of *S. cerevisiae*^19^. In addition, PLP binding results in proper ordering of active pocket residues, leading to the creation of a functional active site with stable conformation^18,20^. Thus, targeting the PLP binding pocket offer an ideal mechanism to inhibit the activity of *w*ALAS since candidates would compete with PLP for binding.

**Figure 6:**
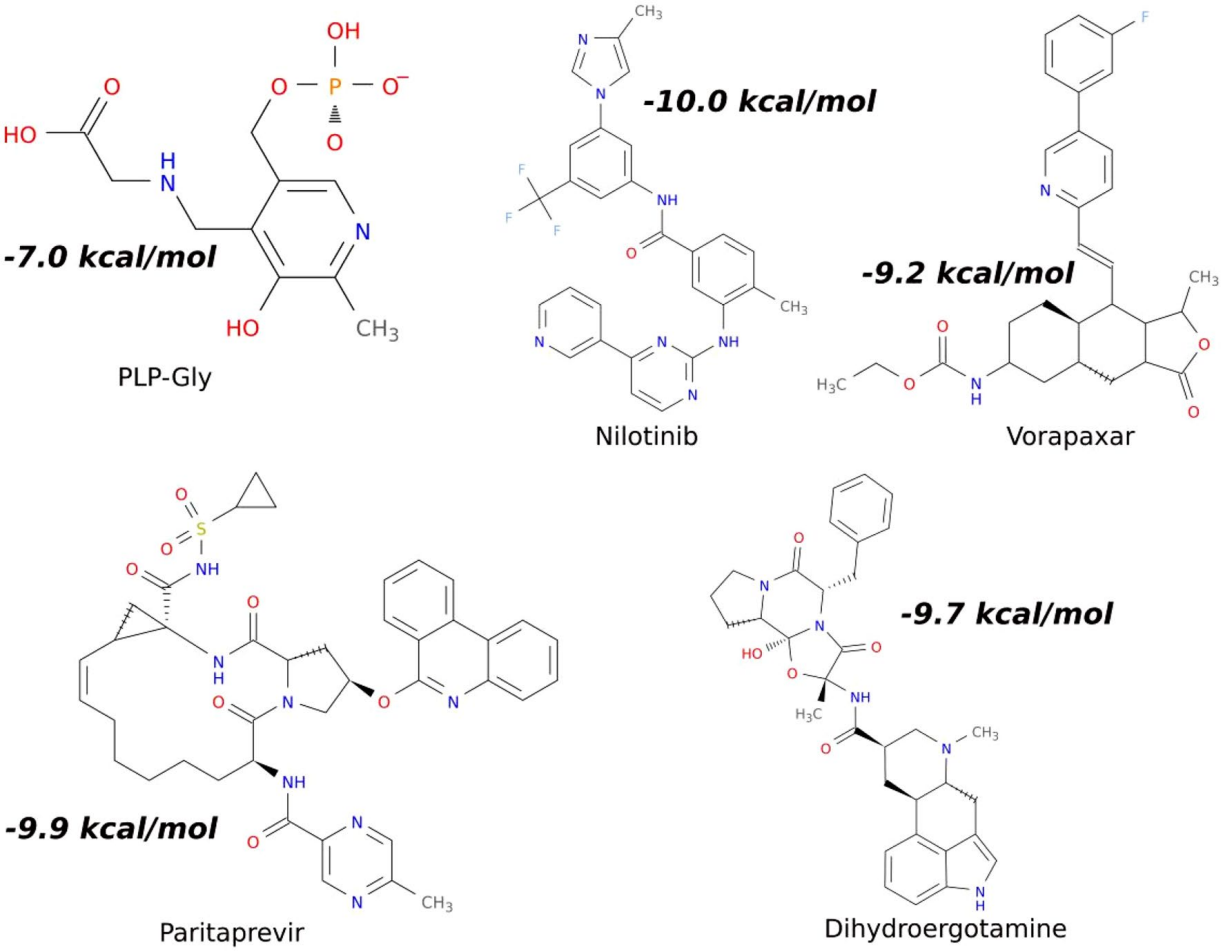
Chemical structures of the selected drug candidates and their autodock binding energies. The binding energy of the natural substrate, PLP-Gly, is also shown.

Accordingly, we considered the ligands that occupied the binding pocket of PLP-Gly as the ideal candidates out of the 21 top-performing hits. This activity resulting in four (4) candidates; ZINC000006716957 (Nilotinib), ZINC000003925861 (Vorapaxar), ZINC0000669678887 (Paritaprevir), and ZINC000003978005 (Dihydroergotamine). The chemical structures and their respective binding energies towards the *w*ALAS binding pocket of the selected candidates and PLP-Gly are shown (Fig. 6). The autodock binding energy can efficiently discriminate between suitable ligands and non-suitable ligands, but does not entirely capture the interactions between the ligand and its receptor^28^. We have used a more accurate calculation algorithm based on molecular simulations, to validate the estimated autodock binding energies.

### MM-PBSA binding energy calculations

The molecular mechanics with Poisson-Boltzmann surface area (MM-PBSA) calculation were used to validate the estimated binding energy from the molecular docking analysis. We run 10 ns simulation for each complex and the trajectories were used for the MM-PBSA binding energy calculations. The g_mmpbsa tool v5.1.2^29^ used for the binding energy calculations does not address the entropic contributions and, therefore, in principle, does not provide the absolute free energy of binding^29^. Nonetheless, the tool is appropriate for determining relative binding energies to compare the interactions between different ligands binding to the same receptor^29^.

The estimated binding energies and the energy terms for nilotinib, vorapaxar, paritaprevir, dihydroergotamine and PLP-Gly against the *w*ALAS are shown in Table 1. Nilotinib showed the highest binding affinity towards *w*ALAS (− 296.6 ± 37.5 kJ/mol), followed by paritaprevir (− 95.7 ± 27.2 kJ/mol), vorapaxar (− 89.1 ± 18.7 kJ/mol) and dihydroergotamine (− 84.7 ± 14.5 kJ/mol).

**Table 1:** MMPBSA analysis of selected drug candidates.

**Ligand**	**MM-PBSA energy (kJ/mol)**				
	**vDW**	**Elec**	**Pol**	**Npol**	**Total**
PLP-Gly	− 104.9 ± 22.5	− 647.7 ± 29.2	729.7 ± 32.8	− 14.8 ± 0.6	− 37.7 ± 33.7
PAR	− 273.6 ± 20.8	− 49.5 ± 15.7	259.5 ± 29.8	− 32.1 ± 1.7	− 95.7 ± 27.2
NIL	− 185.3 ± 20.1	− 1214.1 ± 73.1	1127.7 ± 67.1	− 24.8 ± 1.6	− 296.6 ± 37.5
DHE	− 199.0 ± 20.1	− 47.9 ± 21.5	187.2 ± 18.0	− 25.1 ± 1.9	− 84.7 ± 14.5
VOR	− 229.3 ± 15.6	− 49.2 ± 19.0	215.2 ± 26.2	− 25.9 ± 1.6	− 89.1 ± 18.7

NIL, nilotinib; VOR, vorapaxar; PAR, paritaprevir; DHE, dihydroergotamine.

**Figure 7:**
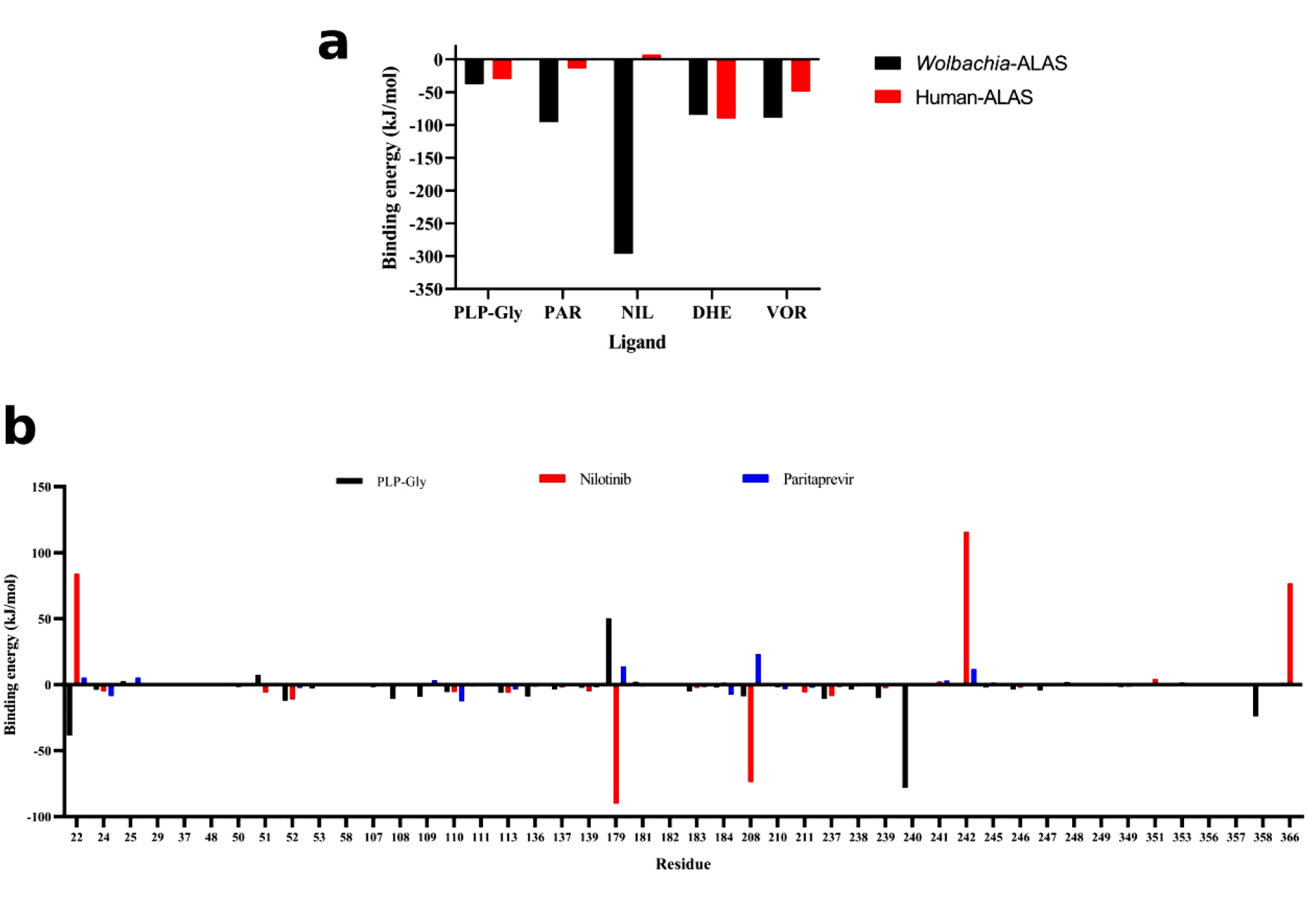
Evaluation of ligand binding energies. (**a**) Comparison of ligand binding energies towards the *w*ALAS and hALAS. NIL = nilotinib, VOR = vorapaxar, PAR = paritaprevir, DHE = dihydroergotamine (**b**) Residual decomposition of binding energy toward the top candidates and PLP-Gly.

The binding affinity of PLP-Gly (− 37.7 ± 33.7 kJ/mol) was comparatively less than the four drug candidates. The human ALAS (hALAS) has different phylogenetic ancestry (Fig. 3b) and distinct biochemical properties compared to the ALAS of filarial endosymbionts^16^. However, some residues are conserved in *w*ALAS and hALAS, especially in the catalytic domain (Fig. 2a). The binding affinities of the candidates towards hALAS were studied through molecular docking and MM-PBSA calculations. The candidates were targeted against the human erythroid-specific 5′-aminolevulinate synthase (PDB ID: 6HRH). The comparison of the MM-PBSA energies of the candidates towards the *w*ALAS and hALAS is summarized in Fig. 7a. Despite the similarity in the ligand binding conformations (occupation of active pockets of both *w*ALAS and hALAS), there are differences in the binding affinities towards the *w*ALAS and hALAS. Vorapaxar and dihydroergotamine had comparable binding affinities towards *w*ALAS and hALAS, although their binding energies are relatively lower than PLP-Gly complexes (Fig. 7a). Based on the structure–function relationship, vorapaxar and dihydroergotamine are unsuitable candidates for investigation as potential therapeutics for filarial infections.

**Figure 8:**
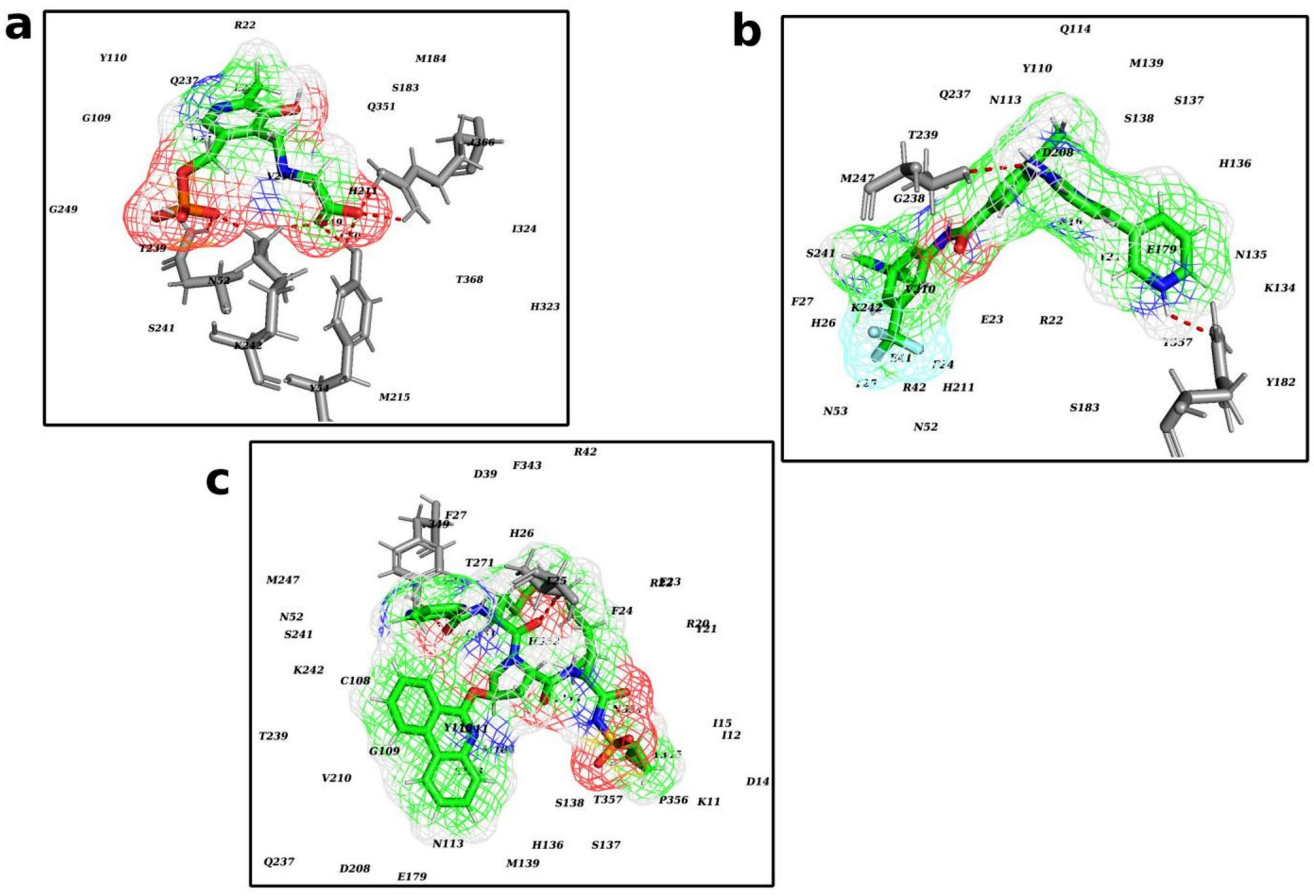
Characterization of the active site residues at the binding site (**a**) PLP-Gly (**b**) Nilotinib (**c**) Paritaprevir.

The binding energies of nilotinib and paritaprevir towards hALAS are 7.2 kJ/mol and − 13.9 kJ/mol, respectively. Comparing the affinity to the hALAS and *w*ALAS, paritaprevir and nilotinib are suitable candidates for filariasis therapy. Moreover, the affinity of paritaprevir and nilotinib are comparably lower than PLP-Gly to hALAS and are therefore unlikely to compete with PLP-Gly for binding sites in hALAS. The total contribution of van der Waals forces in the interaction between PLP-Gly and *w*ALAS is − 104.9 ± 22.5 kJ/mol, compared to paritaprevir (− 273.6 ± 20.8 kJ/mol) and nilotinib (− 185.3 ± 20.1 kJ/mol). The electrostatic contributions to the complexation of PLP-Gly and *w*ALAS is − 647.7 ± 29.2 kJ/mol, compared to paritaprevir (− 49.5 ± 15.7 kJ/mol) and nilotinib (− 1214.1 ± 73.1 kJ/mol). With respect to non-polar interaction energy terms, the contribution to the complexation of PLP-Gly, paritaprevir and nilotinib are − 14.8 ± 0.6 kJ/mol, − 32.1 ± 1.7 kJ/mol and − 24.8 ± 1.6 kJ/mol, respectively. Considering the breakdown of energy terms, paritaprevir and nilotinib have the structural potential to compete with PLP-Gly for binding sites. The drug candidates fit the active pocket and interact with catalytic residues via polar contacts (Fig. 8).

To further explore the protein–ligand interactions at the molecular level, we determined the energy contributions of *w*ALAS residues to the total binding energy. The residue-energy contribution profiles of the complexes are shown in Fig. 7b. The favourable and non-favourable energy contributions of the active residues in the *w*ALAS catalytic pocket are both represented. The active site residues strongly interacting with the PLP-Gly are Arg-22, Asn-52, Cys-108, Asn-237, Thr-239, Leu-240, and Val-358. The strongest favourable energy contributions to nilotinib complexation are from Asn-52, Glu-179, Asp-208 and Gln-237. The active site residues Phe-24, Tyr-110, and Met-184 also contribute strongly to the interactions between paritaprevir and *w*ALAS. Generally, the interactions between *w*ALAS and the ligands are stabilized by minor contributions from many active site residues. The largest non-favourable energy contribution towards the binding of nilotinib and paritaprevir is from Lys-242. This lysine residue is conserved in all ALAS proteins and aid in catalysis by positioning the co-factor appropriately in the active pocket for catalysis to occur^20^. The energy contribution profile also suggests a minor favourable energy contributions from Lys-242 (− 1.2 kJ/mol) towards the binding of PLP-Gly.

### Conformational changes of wALAS upon binding of drug candidates

Next, we investigated the structural dynamics of *w*ALAS upon binding and interactions with the drug candidates. A 200 ns molecular dynamics (MD) simulation of the complexes was performed. The structural changes of the *w*ALAS were characterized by root-mean-square-deviation (RMSD) and root-mean-square fluctuation (RMSF). The RMSF analysis was performed with the last 150 ns of the simulation. We used the conformational profile of the apoenzyme (*w*ALAS without PLP-Gly) as reference to determine the resulting changes upon ligand binding.

**Figure 9:**
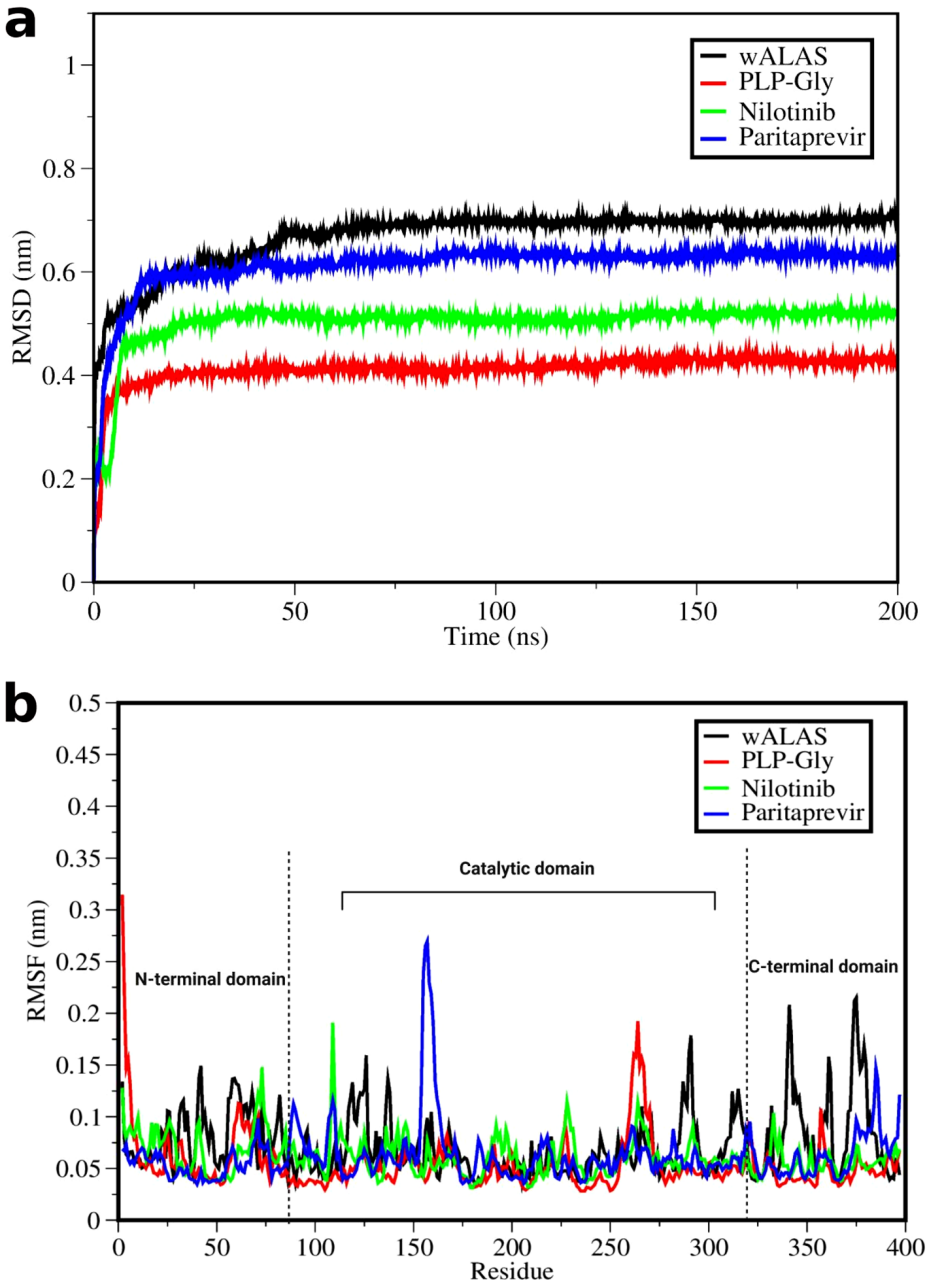
Structural dynamics *w*ALAS upon binding of ligands. (**a**) RMSD profile of the complexes based on 200 ns simulation. (**b**) RMSF profile of *w*ALAS upon binding of the ligands based on the last 150 ns of simulation.

The evolution of the structural stability (RMSD) during the 200 ns MD simulation of the complexes are shown in Fig. 9a. The *w*ALAS recorded an average RMSD of 0.7 nm, which is lower than the RMSD upon binding of PLP-Gly (0.4 nm), paritaprevir (0.6 nm) and nilotinib (0.5 nm). Thus, the protein structure seems to be more stable upon ligand binding when compared to the apoenzyme. The profile of residual fluctuations upon ligand binding shows significant differences in residue behaviour during interactions with the ligands (Fig. 9b). The catalytic domain seems to be less affected when compared to the terminal domains. The structural integrity of the protein is needed for efficient biological activities such as catalysis and dimerization. Our results indicate that ligand binding affects residual flexibility and global dynamics of the protein. It has already been established that paritaprevir and nilotinib have the structural potential to successfully compete with PLP-Gly for binding sites in *w*ALAS. The RMSF profile further suggests that ligand binding may influence the dimerization of the *w*ALAS protomers, considering the difference in residue behaviour during interactions with the ligands.

## Discussion

The *Wolbachia* endosymbiont of filarial worms is now the main therapeutic target for the elimination of filarial diseases^33–35^. The heme biosynthetic pathway represents one of the most important metabolic pathways in the symbiotic relationship between the worm and the *Wolbachia*. The *w*ALAS catalyzes the first step of heme biosynthesis in the *Wolbachia,* which involves the conversion of glycine and succinyl-CoA into aminolevulinate using PLP as co-factor. This pathway in the *Wolbachia* is the sole source of heme for filarial parasites, thus, crucial for the survival of the filarial worm^16,17^. The aim of this study was to identify potential inhibitors of the *w*ALAS using high-throughput screening of FDA-approved drugs and molecular dynamics simulation studies with MM-PBSA calculations.

In our modeled structure of *w*ALAS, the catalytic core lies between the terminal domains with distinct binding pockets for the substrates and co-factors. The structure of *w*ALAS was modeled based on the *Rhodobacter capsulatus* ALAS (PDB ID: 2BWP). Based on comparison with sequences from other filarial parasites and eukaryotic origins, evolutionary relatedness is higher in other filarial ALAS sequences than sequences of eukaryotic origins. Despite the evolutionary divergence, human ALAS shares many conserved residues with the *w*ALAS in the catalytic domain. However, the *w*ALAS and the hALAS have different biochemical properties and sensitivities to particular inhibitors of the heme biosynthesis pathway^16^.

Previous studies have shown that the catalytic mechanism of ALAS involves a Schiff-base pairing of the co-factor and glycine, forming the a catalytically active aldimine (PLP-Gly)^18,19^. Structural elucidation of ALAS has revealed that this Schiff-base interaction involves the co-factor and an active site lysine residue prior to catalysis. Our data show that Lys-242 is the active site residue involved in the Schiff-base linkage to PLP in the *w*ALAS. This Lys-242, together with other residues, form the catalytic core of the PLP-Gly. This highly conserved lysine residue has been associated with the proper positioning of the co-factor for efficient catalysis and contributes to the inter-molecular interactions during catalysis^20^. In the human ALAS, missense mutations in the catalytic core cause hereditary sideroblastic anemia as a result of several phenotypic changes in substrate binding and protein structure integrity^18,19^. The Lys-242 of *w*ALAS could be a potential focus for future mutational studies to better understand the structure–function relationships of the enzyme. Having characterized the binding site of *w*ALAS, we targeted the active pocket of the enzyme for potential binders using over 3200 FDA-approved drugs in a high-throughput virtual screening approach. Preferences were given to candidates that fit the binding pocket of PLP-Gly. Previous studies have highlighted the importance of the co-factor before and during catalysis by *w*ALAS^18–20^. Thus, candidates that fit the PLP-Gly pocket with better affinities have the structural properties to compete with the substrate for binding site. This could be the basis for the inhibition of the candidates. This study identified nilotinib and paritaprevir as suitable anti-*Wolbachia* candidates with the structural potential to successfully compete with the natural substrate of *w*ALAS for the binding site. Nilotinib is a tyrosine kinase inhibitor used for managing chronic myelogenous leukemia^36^. Paritaprevir is an anti-viral drug used as part of a combination regimen for treating chronic Hepatitis C. Paritaprevir targets NS3/4A serine protease of Hepatitis C Virus to inhibit viral replication^37^.

By virtue of their structural appropriateness, nilotinib and paritaprevir bind and fit the active pocket of *w*ALAS and engage active site residues. The estimated ligand binding affinities to the target show that the selected candidates both have comparably higher binding energies towards *w*ALAS than its natural ligand (PLP-Gly). On the basis of competitive inhibitory activity, nilotinib and paritaprevir have advantages over PLP-Gly. Decomposition of binding energy on the basis of residue contribution further confirmed the engagement of active residues by nilotinib and paritaprevir.

The highly conserved Lys-242 contributes with minor favorable energy to the interaction between *w*ALAS with PLP-Gly as already predicted for several ALAS catalysis^18–20^. However, Lys-242 contributes with strong unfavourable energy towards the binding of paritaprevir and nilotinib. In addition to contributions for key active site residues, the complexes were stabilized by minor favourable contributions from residues in the catalytic pocket. The complex between the *w*ALAS and PLP-Gly is structurally stable compared to paritaprevir and nilotinib. However, it seems the binding of paritaprevir and nilotinib results in significant stabilization of the protein system compared to the apoenzyme and leads to substantive differences in residual flexibilities and behavior of protein residues.

Some limitations of the present study should be noted. First, the study did not include experimental validation of the findings. Nonetheless, the approach described in the study ensures that further experimental and clinical studies are less resource-demanding, with a high probability of obtaining the desired results. Second, most structural dynamics and biological activities of proteins occur within timescales of microseconds and milliseconds. In the present study, we investigated protein dynamics and complex stabilities with MD simulations lasting within nanoseconds scales (0–500 ns). The choice of the timescale was informed from the computational power available. Moreover, analyses of protein–ligand interactions and complex dynamics could be accurately informed from MD simulations in nanosecond timescales.

## Conclusion

We have used an in silico drug repurposing approach to find potential anti-Wolbachia drug candidates as therapeutic options for filarial infections. We identified paritaprevir and nilotinib, both FDA-approved medications for managing leukemia and chronic Hepatitis C, respectively, as potential anti-Wolbachia candidates. These drugs have the structural potential to bind and engage active site residues of *w*ALAS, the first enzyme of heme biosynthesis in *Wolbachia*. We hereby propose paritaprevir and nilotinib for experimental validations as anti-Wolbachia agents.
